# The *R* Enantiomer of the Antitubercular Drug PA-824 as a Potential Oral Treatment for Visceral Leishmaniasis

**DOI:** 10.1128/AAC.00722-13

**Published:** 2013-10

**Authors:** Stephen Patterson, Susan Wyllie, Laste Stojanovski, Meghan R. Perry, Frederick R. C. Simeons, Suzanne Norval, Maria Osuna-Cabello, Manu De Rycker, Kevin D. Read, Alan H. Fairlamb

**Affiliations:** Division of Biological Chemistry and Drug Discovery, Wellcome Trust Biocentre, College of Life Sciences, University of Dundee, Dundee, Scotland, United Kingdom

## Abstract

The novel nitroimidazopyran agent (*S*)-PA-824 has potent antibacterial activity against Mycobacterium tuberculosis *in vitro* and *in vivo* and is currently in phase II clinical trials for tuberculosis (TB). In contrast to M. tuberculosis, where (*R*)-PA-824 is inactive, we report here that both enantiomers of PA-824 show potent parasiticidal activity against Leishmania donovani, the causative agent of visceral leishmaniasis (VL). In leishmania-infected macrophages, (*R*)-PA-824 is 6-fold more active than (*S*)-PA-824. Both des-nitro analogues are inactive, underlining the importance of the nitro group in the mechanism of action. Although the *in vitro* and *in vivo* pharmacological profiles of the two enantiomers are similar, (*R*)-PA-824 is more efficacious in the murine model of VL, with >99% suppression of parasite burden when administered orally at 100 mg kg of body weight^−1^, twice daily for 5 days. In M. tuberculosis, (*S*)-PA-824 is a prodrug that is activated by a deazaflavin-dependent nitroreductase (Ddn), an enzyme which is absent in Leishmania spp. Unlike the case with nifurtimox and fexinidazole, transgenic parasites overexpressing the leishmania nitroreductase are not hypersensitive to either (*R*)-PA-824 or (*S*)-PA-824, indicating that this enzyme is not the primary target of these compounds. Drug combination studies *in vitro* indicate that fexinidazole and (*R*)-PA-824 are additive whereas (*S*)-PA-824 and (*R*)-PA-824 show mild antagonistic behavior. Thus, (*R*)-PA-824 is a promising candidate for late lead optimization for VL and may have potential for future use in combination therapy with fexinidazole, currently in phase II clinical trials against VL.

## INTRODUCTION

Visceral leishmaniasis (VL), caused by the protozoan parasite Leishmania donovani, is the second largest parasitic killer after malaria, with more than 200 million people currently at risk from infection. In 95% of cases, death can be prevented by timely and appropriate drug therapy (1); however, current treatment options are far from ideal (2). In India, Bangladesh, and Nepal, the epicenter of VL infection with 60% of the world's reported cases (3), concerted efforts are being made to eliminate the disease by 2015. An important element of this strategy involves active case finding and drug treatment. Antimonial drugs are no longer efficacious in Asia due to drug resistance (2), and the best currently available treatments for anthroponotic VL are miltefosine and liposomal amphotericin B. Undoubtedly, both drugs are vastly superior to previous therapies, but they also have their limitations. The principal drawbacks of miltefosine are its teratogenicity, prolonged treatment regimen, and high resistance potential which limits its usefulness in national elimination programs (4). Problems associated with liposomal amphotericin B include high treatment costs and the need for intravenous administration in primary health care centers (5). In addition, unresponsiveness has been reported in some Sudanese VL patients (6). Thus, there remains an urgent need to strengthen the range of treatment options not only for VL but also for post-kala-azar dermal leishmaniasis (PKDL), which has a 10 to 20% incidence in VL patients from India and 50% in the Sudan following apparently effective drug treatment (7).

In the search for more-effective treatments for the neglected diseases, there has been renewed interest in the chemotherapeutic potential of nitroheterocyclic compounds. This interest largely stems from the success of nifurtimox-eflornithine combination therapy (NECT) for the treatment of the Gambian form of human African trypanosomiasis (HAT). Treatment with NECT, consisting of oral nifurtimox, a nitrofuran drug also used against Chagas' disease, combined with eflornithine infusions, has resulted in cure rates of around 97%, leading to its inclusion on the WHO Essential Medicines List (8). The success of nifurtimox as part of NECT prompted the Drugs for Neglected Disease Initiative (DNDi) to undertake a comprehensive screen of 700 nitroheterocyclic compounds for antiparasitic activity. As a result, the 2-substituted 5-nitroimidazole fexinidazole (Hoe 239), first shown to have antitrypanosomal activity almost 30 years ago (9), was rediscovered. Fexinidazole has now successfully completed phase I clinical trials for HAT and is now in phase II/III trials (10). To date, nitroheterocyclics have not been widely used in the treatment of leishmaniasis; however, in our recent studies we demonstrated that fexinidazole also has potential as a safe and effective oral drug therapy for treatment for the visceral disease (11). In light of these findings, fexinidazole is about to enter phase II clinical trials for the treatment of visceral leishmaniasis in Sudan (http://www.dndi.org).

As a result of our studies with fexinidazole, we searched the literature for other nitroimidazoles which may have potential for use in the treatment of visceral leishmaniasis. In this process, we noted the promising antitubercular drug PA-824 (12, 13). This bicyclic nitroimidazole exhibits potent bactericidal activity against both replicating and nonreplicating Mycobacterium tuberculosis, the causative agent of tuberculosis (TB), and is currently being tested in phase II clinical trials (14–16). Here, we demonstrate that in contrast to the TB structure-activity relationship, it is the *R* enantiomer of PA-824 that displays the greatest *in vitro* potency and has promising leishmanicidal activity *in vivo*. We also demonstrate that the mode of action of (*R*)-PA-824 does not involve the trypanosomatid type I nitroreductase (NTR) that activates fexinidazole and nifurtimox.

## MATERIALS AND METHODS

### Cell lines and culture conditions.

The clonal Leishmania donovani cell line LdBOB (derived from MHOM/SD/62/1S-CL2D) was grown as either promastigotes or axenic amastigotes in medium specific for each developmental stage (17). Amastigotes were cultivated at 37°C in 5% CO_2_, and promastigotes were grown at 26°C. Parasites were cycled between developmental stages after a maximum of 7 passages. Transgenic LdBOB parasites expressing the Leishmania major nitroreductase (LmjF.05.0660) enzyme (11) were cultured under identical conditions.

Procyclic trypomastigotes of Trypanosoma brucei brucei S427 29-13 were grown in SDM-79 medium supplemented with 10% fetal calf serum (FCS) and hemin (100 mg ml^−1^). T. brucei bloodstream forms (S427 Single Marker) were cultured at 37°C in HMI9T medium (18). Trypanosoma cruzi Silvio-X10/7 clone A1 epimastigotes were grown in RTH-FCS at 28°C (19). T. cruzi amastigotes were maintained in Vero cells (African green monkey kidney cells) cultured in minimal Eagle's medium (MEM) supplemented with 1× GlutaMax (Life Technologies) and 10% FCS. HepG2 cells (hepatocyte carcinoma cells) were grown in MEM supplemented with 1× GlutaMax, 1× nonessential amino acids (Life Technologies), and 10% FCS. All mammalian cells were grown at 37°C, 5% CO_2_, in a humidified incubator.

### Chemical synthesis of (*S*)-PA-824 and analogues.

(*S*)- and (*R*)-PA-824 were prepared using a four-step sequence from 2-bromo-4-nitroimidazole (scheme S1). In addition, (*S*)-PA-824 was prepared in a single step as previously described (scheme S2) (20). (*S*)- and (*R*)-Des-nitro-PA-824 were prepared in 5 steps according to the method of Singh et al. (13) (scheme S3). Compound purity was determined by liquid chromatography-mass spectrometry (LC-MS), with all compounds found to be of >95% purity. For *in vivo* experiments, compound purity was further analyzed by ultrahigh-performance liquid chromatography-mass spectrometry (UPLC-MS), with all compounds found to be of ≥99% purity. Optical rotation measurements of (*S*)-PA-824 were in close agreement with published values, confirming a high level of optical purity (21). The optical rotation of (*R*)-PA-824 was equal and opposite to that of the *S* enantiomer, confirming its optical purity. Full experimental protocols and analytical data for all compounds are given in the supplemental material.

### *In vitro* drug sensitivity assays.

To examine the effects of test compounds on growth, triplicate cultures were seeded with 1 × 10^5^ parasites ml^−1^. Parasites (L. donovani promastigotes, T. brucei procyclics, and T. cruzi epimastigotes) were grown in the presence of drug for 72 h, after which 50 μM resazurin was added to each well (0.5 mM in the case of T. cruzi epimastigotes) and fluorescence (excitation of 528 nm and emission of 590 nm) was measured after a further 4 h of incubation. Drug sensitivities of bloodstream-form T. brucei were determined as previously described (22). T. brucei, L. donovani, and T. cruzi epimastigote drug sensitivity data were processed using GRAFIT (version 5.0.4; Erithacus Software) and fitted to a 2-parameter equation, where the data are corrected for background fluorescence, to obtain the effective concentration inhibiting growth by 50% (EC_50_):

$$Y=\frac{100}{1+({\frac{\left[I\right]}{EC_{50}})}^{m}}$$

In this equation, [*I*] represents inhibitor concentration and *m* is the slope factor. Experiments were repeated at least three times, and the data are presented as the weighted mean plus weighted standard deviation (22).

All T. cruzi amastigote and HepG2 drug sensitivity data were processed using the program IDBS ActivityBase. Raw data were converted into percent inhibition through linear regression by setting the high-inhibition control as 100% and the no-inhibition control as 0%. Curve fitting was carried out using the following 4-parameter equation:

$$Y=A+\frac{B-A}{1+({\frac{EC_{50}}{\left[I\right]})}^{m}}$$

In this equation, *A* represents lowest percent inhibition and *B* represents highest percent inhibition. If curve definition was poor, the value of *B* was fixed to 100.

### In-macrophage drug sensitivity assays.

In-macrophage drug sensitivity assays were carried out as previously described (11), using mouse peritoneal macrophages harvested from BALB/c mice.

### In-Vero-cell T. cruzi drug sensitivity assays.

Details of the T. cruzi assay will be published elsewhere (unpublished data). Briefly, T. cruzi trypomastigotes (X10/7) clone A1 were used to infect Vero cells in 384-well plates (Greiner) and then exposed for 72 h to compounds followed by fixation, staining with Hoechst 33342, and evaluation of infection using an Operetta high-content imaging system (PerkinElmer).

### HepG2 assays.

HepG2 cells (2.5 × 10^3^ per well) were plated in 384-well white clear-bottomed plates (Greiner) and cultured in minimal Eagle's medium with 10% FCS for 24 h at 37°C in 5% CO_2_. Compound dilution curves were then added to plates directly using a Labcyte Echo 550 acoustic dispenser (25 nl). After compound addition, plates were incubated for a further 69 h. Resazurin was then added to each well at a final concentration of 50 μM, and fluorescence was measured (excitation of 528 nm and emission of 590 nm) after 1.5 h of incubation.

### Drug combination studies.

For isobologram determinations, (*R*)-PA-824, (*S*)-PA-824, and fexinidazole sulfone were tested in fixed ratios based on their respective EC_50_s. The EC_50_s of each combination were determined after 72 h and plotted as an isobologram (23).

### *In vivo* drug sensitivity.

All animal experiments were approved by the University Ethical Review Committee and performed under the Animals (Scientific Procedures) Act 1986 in accordance with the European Communities Council Directive (86/609/EEC). Groups of female BALB/c mice (5 per group) were inoculated intravenously with approximately 2 × 10^7^ L. donovani amastigotes (LV9; WHO designation MHOM/ET/67/HU3) harvested from the spleen of an infected hamster (24). From day 7 postinfection, groups of mice were treated either with drug vehicle only (orally), with sodium stibogluconate (Pentostam, a gift from GlaxoSmithKline) (15 mg kg of body weight^−1^ subcutaneously), with miltefosine (12 mg kg^−1^ orally), or with either (*R*)- or (*S*)-PA-824 (30 or 100 mg kg^−1^ orally). Miltefosine and sodium stibogluconate were administered once daily for 5 days, with (*R*)- and (*S*)-PA-824 administered twice daily over the same period. Drug dosing solutions were prepared fresh each day, and the vehicle for both enantiomers of PA-824 was 10% (vol/vol) dimethyl sulfoxide (DMSO), 40% polyethylene glycol 400, and 50% deionized water. On day 14 postinfection, all animals were humanely euthanized and parasite burdens were determined by counting the number of amastigotes/500 liver cells (11). Parasite burden is expressed in Leishmania donovani units (LDU), mean number of amastigotes per liver cell × mg weight of liver (25).

### Determination of PA-824 enantiomer exposure in mice after acute oral dosing.

The *R* and *S* enantiomers of PA-824 (50 mg kg^−1^) were orally administered to BALB/c mice. Blood samples (10 μl) were collected from the tail vein of each animal into sample storage tubes (Micronic BV) containing deionized water (20 μl) at defined intervals postdose and stored at −80°C until analysis. The level of each enantiomer in mouse blood was determined by UPLC-MS/MS (20).

### Physicochemical properties and *in vitro* drug metabolism and pharmacokinetic (DMPK) parameters.

The software package StarDrop by Optibrium was used to calculate physical parameters, including log P, molecular weight, and polar surface area (PSA) for (*R*)- and (*S*)-PA-824. The plasma protein binding (PPB) of the enantiomers of PA-824 and its des-nitro analogues was determined by the equilibrium dialysis method (22). The aqueous solubility of the enantiomers of PA-824 was measured using laser nephelometry (BMG Labtech nephelometer) following serial dilution of DMSO stocks into deionized water to give a final concentration range of 12 to 250 μM, with a final DMSO concentration of 2.5%. The amount of laser scatter caused by insoluble particulates (relative nephelometry units) was plotted against compound concentration using a segmental regression fit, with the point of inflection being quoted as the aqueous solubility. To measure the intrinsic clearance (CL_int_), each enantiomer of PA-824 (0.5 μM) was incubated with female CD1 mouse liver microsomes (Xenotech LLC; 0.5 mg ml^−1^ in 50 mM potassium phosphate buffer, pH 7.4) and the reaction was started by the addition of excess NADPH (8 mg ml^−1^). At time zero and then at 3, 6, 9, 15, and 30 min, an aliquot (50 μl) of the incubation mixture was mixed with acetonitrile (100 μl) to stop the reaction. A structural analogue was then added to all samples as an internal standard, the samples were centrifuged to sediment precipitated protein, and the plates were sealed prior to UPLC-MS/MS analysis using a Quattro Premier XE spectrometer (Waters Corporation, USA). XLfit (IDBS, United Kingdom) was used to calculate the exponential decay and consequently the rate constant (*k*) from the ratio of peak area of test compound to internal standard at each time point. CL_int_ was then calculated using the following equation: CL_int_ (ml min^−1^ [g liver]^−1^) = *k* × *V* × microsomal protein yield, where *V* (ml mg protein^−1^) is the incubation volume per mg protein added and microsomal protein yield is taken as 52.5 mg protein per g liver. Verapamil (0.5 μM) was used as a positive control to confirm acceptable assay performance.

## RESULTS

### *In vitro* sensitivity of L. donovani and related parasites to (*S*)- and (*R*)-PA-824.

The promise shown by fexinidazole as an oral treatment for visceral leishmaniasis prompted us to investigate the possibility that other nitroheterocyclic compounds may also have potential in treating this disease. With this in mind, the antitubercular clinical candidate (*S*)-PA-824, currently in phase II development by the TB Alliance, was synthesized and assessed for antileishmanial activity. The potency of (*S*)-PA-824 was determined *in vitro* against L. donovani (LdBOB) promastigotes and against intracellular amastigotes in peritoneal mouse macrophages. (*S*)-PA-824 showed antileishmanial activity against both developmental stages of the parasite, with EC_50_s of 0.9 ± 0.1 and 4.9 ± 0.3 μM against promastigotes and amastigotes, respectively (Table 1). However, (*S*)-PA-824 was largely inactive against the mammalian stages of T. brucei and T. cruzi, with EC_50_s of >30 μM in both cases. (*S*)-PA-824 was also found to be inactive (EC_50_, >50 μM) in a counterscreen against the mammalian cell line HepG2.

**Table 1:**
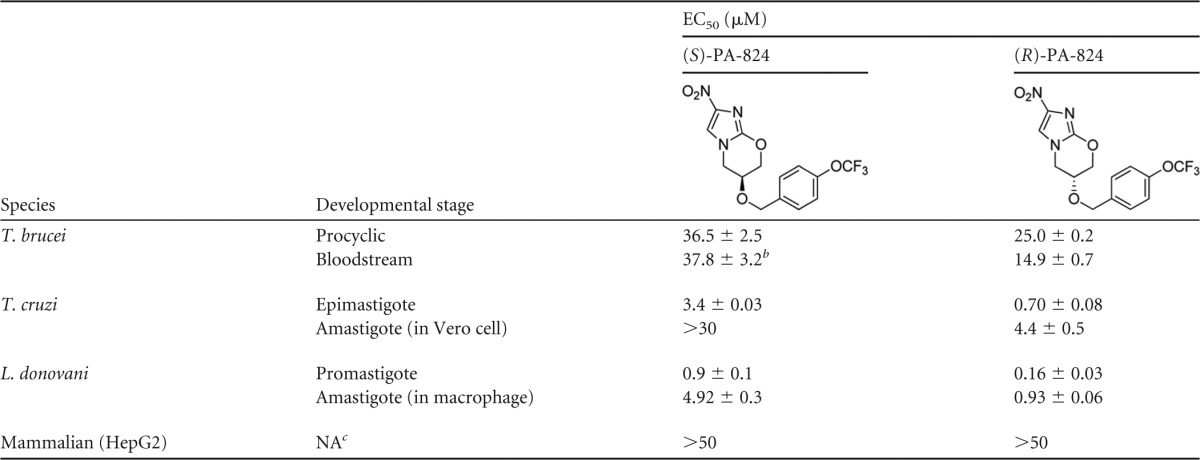
EC_50_s for PA-824 enantiomers against representative trypanosomatids^*a*^

a Results are the weighted means and standard errors of at least three independent experiments, except for HepG2 (*n* = 2).

b Previously determined value (20).

c NA, not applicable.

The *R* enantiomer of PA-284 has been shown to have little or no activity against Mycobacterium tuberculosis (MIC, >100 μM) (26); however, as part of a limited hit expansion program, (*R*)-PA-824 was synthesized and its antileishmanial activity was assessed. In direct contrast to that seen in M. tuberculosis, (*R*)-PA-824 proved to be a ∼5-fold-more potent inhibitor of L. donovani growth *in vitro* than the *S*-enantiomer candidate, with EC_50_s of 0.16 ± 0.03 and 0.9 ± 0.1 μM against promastigotes and intracellular amastigotes, respectively (Table 1). Indeed, (*R*)-PA-824 also showed slightly improved activity against the mammalian stages of T. brucei and T. cruzi while remaining inactive against the mammalian cell line HepG2 (EC_50_, >50 μM).

### Pharmacological profiling of (*S*)- and (*R*)-PA-824 and (*R*)-des-nitro-PA-824 *in vitro*.

A number of studies have reported that (*S*)-PA-824 is well tolerated in mice and can reduce M. tuberculosis infection levels after oral dosing (27, 28). To determine if (*R*)-PA-824 was similarly suitable for *in vivo* studies, the plasma protein binding (PPB), aqueous solubility, and CL_int_ were measured and compared to those of the *S* enantiomer. Both enantiomers had good aqueous solubility (>250 μM) and similar low mouse PPB, with the fractions unbound for (*S*)- and (*R*)-PA-824 being 0.21 and 0.22, respectively. The fraction unbound for the (*R*)-des-nitro-PA-824 is 0.45. (*R*)-PA-824 shows higher mouse microsomal stability than does the *S* enantiomer (CL_int_ = 0.65 versus 1.6 ml min^−1^ g^−1^). The calculated values for the partition coefficient, log P (2.7), and polar surface area (91 Å^2^) were identical for the two enantiomers.

### *In vivo* pharmacokinetic properties of (*S*)- and (*R*)-PA-824.

**Fig 1:**
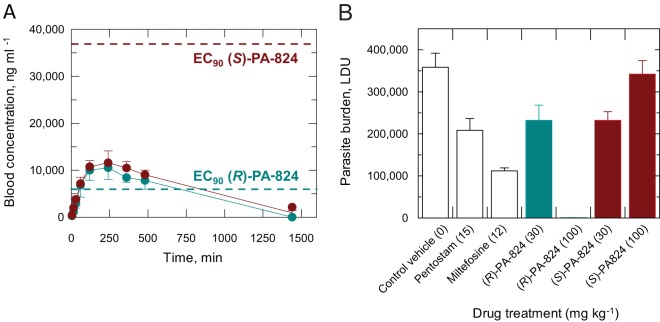
*In vivo* pharmacokinetic properties and efficacy of PA-824 in mice. (A) Blood concentrations of (*R*)- and (*S*)-PA-824 following oral dosing at 50 mg kg^−1^. The EC_90_ values of both enantiomers for L. donovani (strain LV9) cultured in *ex vivo* mouse macrophages and corrected for plasma protein binding are shown as dashed lines. (*S*)-PA-824 data are shown in maroon, and (*R*)-PA-824 data are shown in teal. Data are the means and standard deviations from three mice. (B) Effects of drug treatment on the parasite burden of mice infected with L. donovani. Mice (five per group) were dosed with drug vehicle (orally), sodium stibogluconate (Pentostam) (subcutaneously), miltefosine (orally), or (*S*)-PA-824 or (*R*)-PA-824 (both orally) for 5 days. Miltefosine and sodium stibogluconate were administered once daily, and PA-824 enantiomers were administered twice daily.

Blood levels of both enantiomers of PA-824 were determined after oral dosing to establish a suitable dosing regimen for VL efficacy studies. Mice dosed with (*S*)- or (*R*)-PA-824 at 50 mg kg^−1^ showed a maximum concentration in blood after 4 h of 11,600 or 10,500 ng ml^−1^, respectively (Fig. 1A). Thereafter, blood concentrations decreased [elimination half-life (*t*_1/2_), ∼5 h and 2 h for (*S*)- or (*R*)-PA-824, respectively], reaching undetectable levels at a time point between 8 and 24 h for (*R*)-PA-824. At 24 h, (*S*)-PA-824 still had a blood concentration of 900 ng ml^−1^.

(*R*)-PA-824 has an EC_50_ of 0.93 μM for L. donovani cultured in macrophages (Table 1) with a Hill slope of 1.7, giving an EC_90_ value of 3.4 μM (equivalent to 1,200 ng ml^−1^). After adjusting for plasma protein binding [(*R*)-PA-824 fraction unbound = 0.22], the estimated blood concentration required to exceed the EC_90_ becomes 5,600 ng ml^−1^. Comparison with the measured levels (Fig. 1A) shows that (*R*)-PA-824 blood levels exceed the EC_90_ 1 h after dosing and remain above the EC_90_ for at least 7 h. Thus, 100 mg kg^−1^ twice daily was predicted to provide adequate exposure for (*R*)-PA-824. Although (*S*)- and (*R*)-PA-824 reach comparable blood levels, the lower potency of (*S*)-PA-824 (EC_90_ = 22 μM) results in a maximal free concentration below EC_90_ (37,000 ng ml^−1^) after oral dosing at 50 mg kg^−1^ (Fig. 1A).

### *In vivo* sensitivity of L. donovani to the enantiomers of PA-824.

The efficacy of both (*R*)- and (*S*)-PA-824 was assessed in a mouse model of visceral leishmaniasis. Seven days following infection with L. donovani LV9 *ex vivo* amastigotes, groups of BALB/c mice were dosed orally with (*R*)- or (*S*)-PA-824 (30 or 100 mg kg^−1^) twice daily and for five consecutive days. Fourteen days following inoculation, parasite burdens in the livers of infected mice were determined. Current antileishmanial chemotherapies sodium stibogluconate (15 mg kg^−1^) and miltefosine (12 mg kg^−1^) were included in the study for comparison. Both enantiomers of PA-824 were extremely well tolerated by mice throughout the study, with no overt signs of toxicity. Mice dosed twice daily at 30 mg kg^−1^ with (*R*)- or (*S*)-PA-824 demonstrated similar efficacies, with both enantiomers suppressing infection in the murine model by approximately 35% compared to untreated controls (Fig. 1B). Administering an increased dose of 100 mg kg^−1^did not improve the efficacy of (*S*)-PA-824 *in vivo*, with L. donovani infection in this group of mice at levels comparable to those seen in drug-free animals. An earlier *in vivo* study, with mice given a range of doses down to 0.4 mg kg^−1^, demonstrated the apparent absence of a dose-dependent leishmanicidal effect with the *S* enantiomer of PA-824 (see Fig. S1 in the supplemental material). Interestingly, these findings mirror those seen in clinical trials of (*S*)-PA-824 in humans with tuberculosis (29). In stark contrast, treatment with (*R*)-PA-824 at 100 mg kg^−1^ effectively cured the murine model of infection, suppressing infection by 99.9%. Dosing with (*R*)-PA-824 at this level proved to be superior to treatment with current front-line drugs sodium stibogluconate (41.9% suppression) and miltefosine (68.7% suppression), underlining the therapeutic potential of this nitroimidazo-oxazine compound.

### (*R*)-PA-824 is not activated by L. major type I nitroreductase.

**Fig 2:**
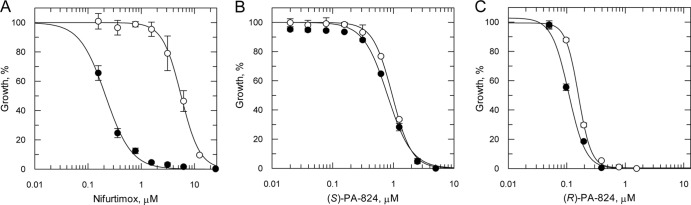
Susceptibility of NTR-overexpressing LdBOB promastigotes to nifurtimox, (*R*)-PA-824, and (*S*)-PA-824. EC_50_s were determined for nifurtimox (A), (*S*)-PA-824 (B), and (*R*)-PA-824 (C) against WT (open circles) and NTR-overexpressing (closed circles) parasites. EC_50_s of 5.6 ± 0.16 and 0.2 ± 0.09 μM for nifurtimox, 0.96 ± 0.02 and 0.8 ± 0.06 μM for (*S*)-PA-824, and 0.16 ± 0.003 and 0.11 ± 0.005 μM for (*R*)-PA-824 were determined against WT and NTR-overexpressing cell lines, respectively. Data are the means of triplicate measurements.

(*S*)-PA-284 is believed to function as a prodrug which requires bioreductive activation prior to exhibiting antitubercular activity (13, 30). In M. tuberculosis, this reduction is catalyzed by an unusual deazaflavin (F_420_)-dependent nitroreductase (Ddn). In the absence of a Ddn homologue in the trypanosomatids, we hypothesized that reduction of (*R*)- and (*S*)-PA-824 in L. donovani may be catalyzed by the NADH-dependent nitroreductase, which has homology to bacterial type I nitroreductases and has already been shown to play a crucial role in the activation of fexinidazole and its metabolites (11). To determine whether reduction by this nitroreductase was central to the mechanism of action of PA-824, the potency of both enantiomers was determined against wild-type (WT) parasites and parasites overexpressing the nitroreductase. Increased concentrations of nitroreductase in these transgenic parasites were confirmed by a 26-fold increase in their sensitivity to nifurtimox (Fig. 2A), a nitrofuran drug known to undergo two-electron reduction by nitroreductase (31). However, overexpression of nitroreductase in promastigotes did not significantly alter their sensitivity to either (*S*)-PA-824 (EC_50_ of 0.96 ± 0.02 and 0.8 ± 0.04 μM for wild-type and transgenic parasites, respectively) or (*R*)-PA-824 (EC_50_ of 0.16 ± 0.002 and 0.11 ± 0.005 μM for wild-type and transgenic parasites, respectively) (Fig. 2B and C). These findings suggest that this nitroreductase does not play a crucial role in the activation of PA-824 in L. donovani.

### *In vitro* sensitivity of L. donovani to des-nitro-PA-824.

To determine whether the nitro group was important for the antileishmanial activity of PA-824, the potencies of (*R*)- and (*S*)-des-nitro-PA-824 were determined against L. donovani promastigotes and intracellular amastigotes. In both assays, both enantiomers of des-nitro-PA-824 were found to be inactive at the highest concentration tested (50 μM), suggesting that the nitro group either plays a crucial role in the mechanism of action of PA-824 or mediates the binding between PA-824 and its molecular target(s) in L. donovani.

### (*R*)-PA-824 combination therapy.

**Fig 3:**
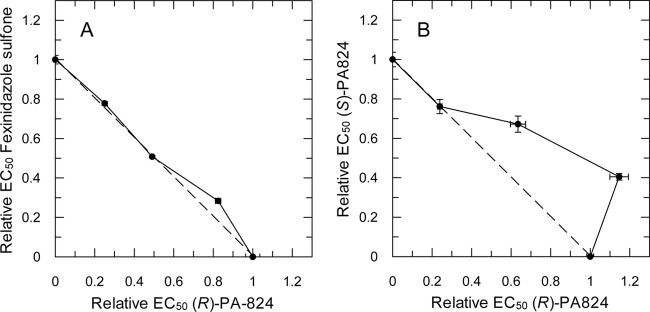
Relative EC_50_s for combinations of (*R*)-PA-824 with fexinidazole sulfone or (*S*)-PA-824. Isobolograms show the relative EC_50_s obtained with combinations of (*R*)-PA-824 with fexinidazole sulfone (A) or (*S*)-PA-824 (B) against promastigotes of the LdBOB strain of L. donovani. (A) Absolute EC_50_s: (*R*)-PA-824, 0.18 μM; fexinidazole sulfone, 8.8 μM. (B) Absolute EC_50_s: (*R*)-PA-824, 0.10 μM; (*S*)-PA-824, 0.74 μM. Data are the weighted means ± standard errors of duplicate cultures.

Recent studies have illustrated the potential of drug combinations in the treatment of visceral leishmaniasis (32). Multidrug therapy is perceived to have several advantages over conventional monotherapy, such as the potential for synergistic activity resulting in increased drug efficacy and the reduced probability of drug resistance emerging. With this in mind, we assessed the *in vitro* interactions of (*R*)-PA-824 in combination with fexinidazole sulfone and (*S*)-PA-824 against L. donovani promastigotes using the fixed-ratio isobologram method (Fig. 3). Isobologram analysis of the potency of various (*R*)-PA-824 and fexinidazole sulfone combinations against promastigotes revealed a near-linear relationship, suggesting that these two compounds act additively in combination (Fig. 3A). However, combinations of (*R*)- and (*S*)-PA-824 demonstrated a more complex relationship. As the proportion of (*R*)-PA-824 in the mixture increases, the two enantiomers become progressively more antagonistic. A second experiment gave an identical pattern.

### PA-824-mediated cell killing.

**Fig 4:**
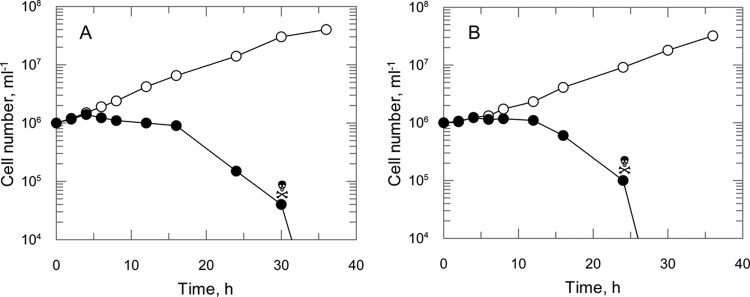
Cytocidal effects of PA-824 enantiomers on L. donovani promastigotes. The *S* (A) and the *R* (B) enantiomers of PA-824 were added to early-log-phase cultures of LdBOB promastigotes (∼1 × 10^6^ ml^−1^) at concentrations equivalent to 10 times their respective EC_50_s. At intervals, the cell densities were determined, and samples of culture (500 μl) were removed, washed, and resuspended in fresh culture medium in the absence of drug. The viability of washed parasites was monitored for up to 72 h following removal from drug exposure, and the point of irreversible drug toxicity was determined (skull and crossbones). Open circles, no inhibitor; filled circles, presence of drug.

To determine whether PA-824 was cytostatic or cytotoxic, we incubated mid-log-phase promastigotes with either (*S*)- or (*R*)-PA-824 at concentrations equivalent to 10 times their respective EC_50_s (Fig. 4). In both cases, growth of drug-treated cultures ceased almost immediately, with cell numbers beginning to decline after 12 h. In cultures treated with (*S*)-PA-824, no intact parasites were visible at 36 h (Fig. 4A). However, cell death was more rapid in parasites treated with the *R* enantiomer, with no live parasites remaining at 24 h (Fig. 4B). To determine the actual point where treated cells completely lost viability, we washed and subcultured parasites at defined intervals without drug. No parasites could be recovered at 30 h following treatment with (*S*)-PA-824. In comparison, the more rapid cytotoxic effect of (*R*)-PA-824 resulted in parasites losing viability by 24 h. The fact that both enantiomers of PA-824 are leishmanicidal rather than cytostatic is highly beneficial, since successful drug therapy with either compound is unlikely to depend on a fully functional patient immune response (33).

## DISCUSSION

The repurposing of existing drugs and clinical candidates for the treatment of neglected diseases of poverty offers a cost-efficient approach toward identifying lead compounds for these unprofitable indications. This strategy has proven successful in the pursuit of treatments for VL, with the current oral therapy miltefosine and the clinical candidate fexinidazole both originally developed for other indications. The potential for the nitroimidazole fexinidazole to treat HAT (34), VL (11), and Chagas' disease (35) is also an example of the current resurgence of interest in the potential of nitro compounds as therapeutics. Encouraged by our findings with fexinidazole (11), now entering phase II clinical trials against VL in Africa, we decided to investigate the potential of other nitroaromatics against this disease. A search of the literature revealed that the antitubercular nitroimidazole (*S*)-PA-824 had been reported to possess an undefined level of activity against L. donovani axenic amastigotes (36). Both M. tuberculosis and L. donovani reside in macrophages *in vivo*, and so the investigation of antitubercular agents was deemed a particularly appropriate starting point in the search for potential VL treatments.

In the current study, we have shown that (*S*)-PA-824 is active against L. donovani intracellular amastigotes at a potency comparable to that of fexinidazole sulfone (EC_50_, 4.9 μM versus 5.3 μM), an active metabolite of the VL clinical candidate fexinidazole. In contrast to the structure-activity relationship for M. tuberculosis, where the *S* enantiomer of PA-824 is >100-fold more active than the *R* enantiomer (26), we found that (*R*)-PA-824 was 5-fold more potent than the *S* enantiomer in the L. donovani intramacrophage assay. Similarly, (*R*)-PA-824 was also more active against the disease-relevant stages of T. brucei and T. cruzi but showed no measurable activity in a mammalian cell counterscreen. Both enantiomers of PA-824 were also found to be leishmanicidal *in vitro* with good selectivity, good microsomal stability, good aqueous solubility, and low plasma protein binding.

Both enantiomers were orally bioavailable and were well tolerated in mice. The pharmacokinetic profiles of the two compounds were similar, but only the *R* enantiomer was predicted to show complete coverage above the EC_90_ for the entire duration of treatment (100 mg kg^−1^, orally, twice a day for 5 days). Indeed, under this treatment regimen (*R*)-PA-824 almost completely cleared L. donovani parasites from the livers of infected mice. When either (*R*)- or (*S*)-PA-824 was administered at 30 mg kg^−1^, there was only a moderate reduction in the observed parasite burden (∼35%). Although we have not established linearity of exposure with dose for either compound, this modest efficacy is consistent with the anticipated sub-EC_90_ free inhibitor blood concentration. Surprisingly, (*S*)-PA-824 becomes less efficacious when administered at 100 mg kg^−1^. We hypothesize that this unexpected reduction in potency may be formulation related.

The target product profile (http://www.dndi.org/) for VL calls for an oral drug that can be used to treat immunosuppressed patients. Significantly, (*R*)-PA-824 is cytocidal rather than cytostatic, which is advantageous because treatment with this compound would not be dependent on the patient having a fully functional immune response. In addition, (*R*)-PA-824 is orally available. Collectively, our study indicates that (*R*)-PA-824 represents a strong lead compound with potential for development as a much-needed oral treatment for VL. However, it is important to note that the *S*, not the *R*, enantiomer of PA-824 has advanced into clinical trials for the treatment of TB. With this in mind, (*R*)-PA-824 should be considered a worthy candidate for lead optimization and not a classical drug-repurposing project.

Overexpression of the L. major nitroreductase did not alter the potency of (*R*)- or (*S*)-PA-824 against L. donovani promastigotes. This demonstrates that (*R*)- and (*S*)-PA-824 are not activated by the same type I nitroreductase responsible for the bioactivation of the antileishmanial nitroimidazole fexinidazole. This is consistent with the observation that lab-generated strains of nifurtimox-resistant T. brucei (nifurtimox is activated by a type I NTR) were cross resistant to fexinidazole but showed no change in sensitivity to (*S*)-PA-824 (20). However, both (*R*)- and (*S*)-des-nitro-PA-824 were found to be inactive against L. donovani, suggesting that the nitro group does play a key role in the antileishmanial activity of this compound series. This result allows for the possibility that (*R*)-PA-824 relies on nitro group reduction to exert its antileishmanial activity. L. donovani does not possess a homologue of the M. tuberculosis deazaflavin nitroreductase (Ddn), which specifically reduces (*S*)-PA-824, but not the *R* enantiomer (26), and since Leishmania type I NTR cannot activate (*R*)-PA-824, this putative bioreduction would need to be mediated by an as-yet-unidentified nitroreductase. Alternatively, it is entirely possible that (*R*)-PA-824 exerts its activity by inhibiting the function of an essential protein. If that were the case, then the nitro substituent could mediate an important binding interaction with the target protein. It is known that nitro groups can play a role in small-molecule protein binding by forming hydrogen bonds, by polarizing the π system of an aromatic ring, or by reducing the basicity of an imidazole ring (37). We are currently developing LC-MS/MS methods in order to monitor the metabolism of PA-824 *in vitro*. In preliminary experiments, we have established that PA-824 disappears over time in supernatants of L. donovani cultures, suggesting that drug metabolism may be occurring. Further investigations aimed at determining the mode of action of (*R*)-PA-824 in Leishmania are under way.

The target product profile for VL also stipulates that new compound entities should be suitable for combination therapy. Our studies suggest that (*R*)-PA-824 and fexinidazole do not share a mechanism of action involving the Leishmania type I nitroreductase, despite both being nitroimidazoles. Therefore, a single mutation is less likely to confer resistance to both compounds. Combination studies demonstrated that fexinidazole and (*R*)-PA-824 have a pharmacological additive effect against L. donovani promastigotes, and so the two compounds could potentially be developed as an oral combination therapy for VL. Interestingly, a combination analysis between (*R*)- and (*S*)-PA-824 revealed that the two enantiomers were mildly antagonistic. Without knowing how these compounds exert their antileishmanial activity, it is difficult to draw conclusions from this result. However, it can be postulated that the less-active *S* enantiomer is competing with the *R* enantiomer for a common uptake mechanism, or for the postulated activating enzyme or other unknown target.

In conclusion, we have identified bicyclic nitroimidazoles as potential therapeutics against VL with a mode of action that is distinct from that of fexinidazole. Our findings suggest that (*R*)-PA-824, or analogues thereof, are suitable oral development candidates for the treatment of VL, with the potential for use in combination with fexinidazole.

## Supplementary Material

Supplemental material
