# Supplementary material for: The R Enantiomer of the Antitubercular Drug PA-824 as a Potential Oral Treatment for Visceral Leishmaniasis
Source: Antimicrob Agents Chemother. 2013 Oct;57(10):4699–706. doi: 10.1128/AAC.00722-13 (PMC3811480; doi:10.1128/AAC.00722-13)
Supplement: Supplemental material [file supp_57_10_4699__index.html]

Supplemental material 

# The *R* Enantiomer of the Antitubercular Drug PA-824 as a Potential Oral Treatment for Visceral Leishmaniasis

## Supplemental material

**Files in this Data Supplement:**

- Supplemental file 1 -

  Supplemental chemistry methods and synthetic schemes S1 to S3. Figure S1, effects of drug treatment on the parasite burden of mice infected with *L. donovani*.

  PDF, 229K
