# Supplementary material for: The R Enantiomer of the Antitubercular Drug PA-824 as a Potential Oral Treatment for Visceral Leishmaniasis
Source: Antimicrob Agents Chemother. 2013 Oct;57(10):4699–706. doi: 10.1128/AAC.00722-13 (PMC3811480; doi:10.1128/AAC.00722-13)
Supplement: Supplemental material [file AAC.00722-13_zac010132168so1.pdf]

SUPPLEMENTAL MATERIAL

The *R*-enantiomer of the anti-tubercular drug PA-824 as a potential treatment for visceral leishmaniasis

Stephen Patterson, Susan Wyllie, Laste Stojanovski, Meghan R. Perry, Frederick R.C. Simeons, Suzanne Norval, Maria Osuna-Cabello, Manu De Rycker, Kevin D. Read and Alan H. Fairlamb

Files in this Data Supplement:

Supplementary chemistry methods and synthetic schemes S1-S3.

Figure S1, effects of drug treatment on the parasite burden of mice infected with *L. donovani*.

SUPPLEMENTARY METHODS

General Chemistry.

Chemicals and solvents were purchased from the Aldrich Chemical Co., Fluka, ABCR, VWR, Acros, Fisher Chemicals, and Alfa Aesar and were used as received. Air and moisture sensitive reactions were carried out under an inert atmosphere of argon. Analytical thin-layer chromatography (TLC) was performed using pre-coated TLC plates (layer 0.20 mm silica gel 60 with fluorescent indicator UV254, from Merck). Developed plates were air-dried and analyzed under a UV lamp (UV254/365 nm). Flash column chromatography was performed using prepacked silica gel cartridges (230-400 mesh, 40-63 µm, from SiliCycle) using a Teledyne ISCO CombiFlash Companion, or CombiFlash Rf. ^{1}H NMR, ^{13}C NMR, ^{19}F NMR, and 2D-NMR spectra were recorded on a Bruker Avance DPX 500 spectrometer (^{1}H at 500.1 MHz, ^{13}C at 125.8 MHz, ^{19}F at 470.5 MHz), or a Bruker Avance DPX 300 (^{19}F at 282 MHz). Chemical shifts (δ) are expressed in ppm recorded using the residual solvent as the internal reference in all cases. Signal splitting patterns are described as singlet (s), doublet (d), triplet (t), quartet (q), multiplet (m), broad (br), or a combination thereof. Coupling constants (*J*) are quoted to the nearest 0.5 Hz. LC-MS analyses were performed with either an Agilent HPLC 1100 series connected to a Bruker Daltonics MicrOTOF or an Agilent Technologies 1200 series HPLC connected to an Agilent Technologies 6130 quadrupole LC/MS, where both instruments were connected to an Agilent diode array detector. LCMS chromatographic separations were conducted with either a Waters XBridge C18 column, 50 mm × 2.1 mm, 3.5 µm particle size, or Waters XSelect C18 column, 30 mm × 2.1 mm, 2.5 µm particle size; mobile phase, water/acetonitrile + 0.1% HCOOH, or water/acetonitrile + 0.1% NH_3. High-resolution electrospray measurements were performed on a Bruker Daltonics MicrOTOF mass spectrometer. Preparative HPLC separations were performed with a Gilson HPLC (321 pumps, 819 injection module, 215 liquid handler/injector) connected to a Gilson 155 UV/vis detector. HPLC chromatographic separations were conducted using a Waters XBridge C18 column, 19 × 100 mm, 5 µm particle size; mobile phase, water/acetonitrile + 0.1% NH_3, or HCOOH. Microwave-assisted chemistry was performed using a Biotage Initiator microwave synthesizer.

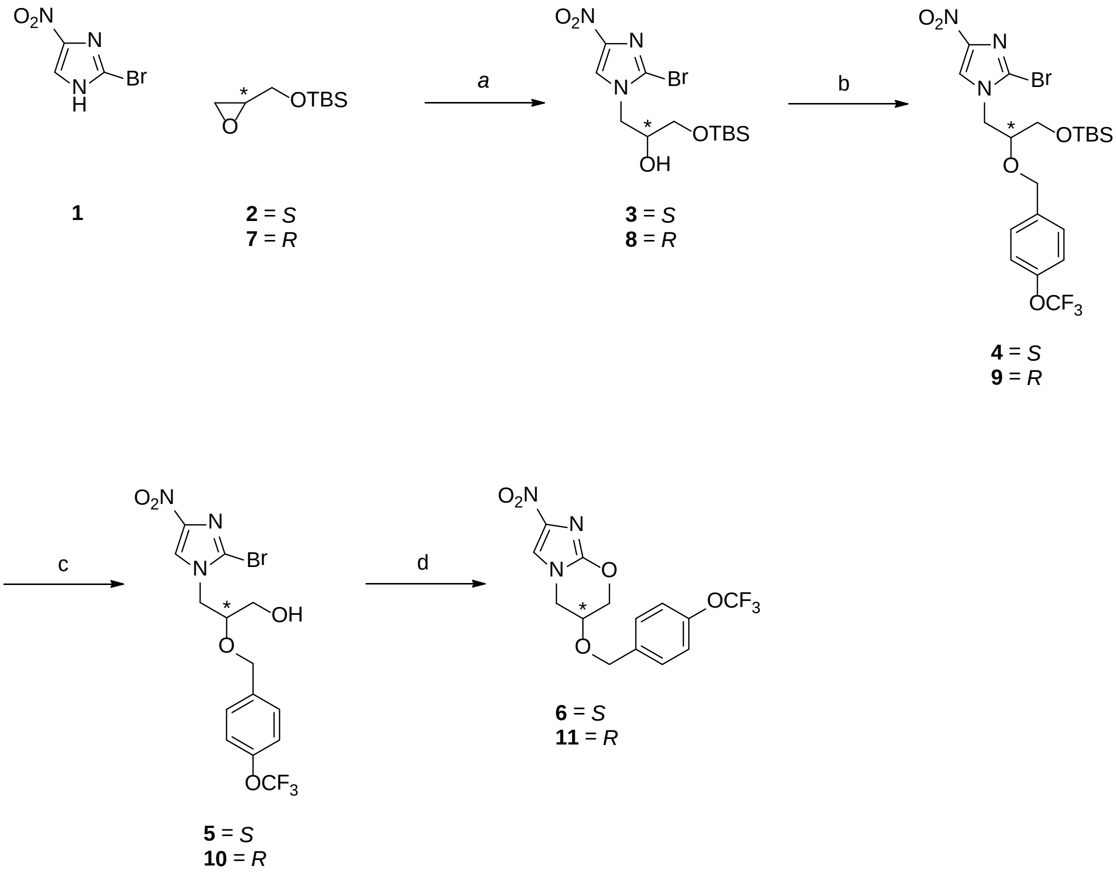

Scheme S1: **Four step synthesis of (*S*)- and (*R*)-PA-824.** Reagents and conditions: (a) K_2CO_3, EtOH, 78°C, 3-10 h; (b) NaH, TBAI, *p*-OCF_3-BnBr, DMF, -50°C →RT, 16 h; (c) TBAF, THF, RT, 45 min; (d) NaH, DMF, 0°C →RT, 2 h.

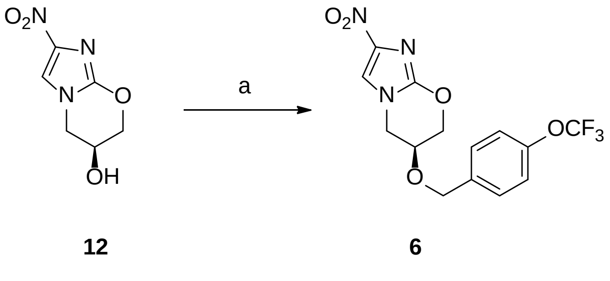

Scheme S2: One step synthesis of (*S*)-PA-824. Reagents and conditions: (a) NaH, TBAI, *p*-OCF_3-BnBr, DMF, -50°C →RT, 16.5 h.

Synthesis of (*S*)-PA-824 (6).

(*S*)-PA-824 (**6**) was either prepared in five steps (Scheme S1), or in a single step from commercially available **12** when available (Scheme S2).

(*S*)-1-(2-bromo-4-nitro-1H-imidazol-1-yl)-3-((*tert*-butyldimethylsilyl)oxy)propan-2-ol (3) (1).

Neat *tert*-butyl(dimethyl)silyl (2*S*)-2-oxiranylmethyl ether (**2**) (3.77 g, 20 mmol), was added to a solution of 2-bromo-4-nitro-imidazole (**1**) (3.84 g, 20 mmol) and K_2CO_3 (387 mg, 2.8 mmol) in anhydrous EtOH (100 ml) and stirred at 78°C for 10 h, after which the solvent was removed under reduced pressure. The resultant crude mixture was partitioned between EtOAc and satd. aq. $NaHCO_3$ (1:1, 400 ml), the layers separated and the aq. extracted with EtOAc (3×200 ml). The combined EtOAc layers were dried over $MgSO_4$, filtered and the solvent removed under reduced pressure. The crude product was purified by silica column chromatography (120 g silica, EtOAc/Hexane 0:100 → 75:25) to give the title compound as a white solid (2.36 g, 31%). ^{1}H NMR (500 MHz, $CDCl_3$): δ 7.97 (s, 1H, ArH), 4.25-4.20 (m, 2H, CH_2), 4.02-3.97 (m, 2H, CH & C*H*H), 3.69 (dd, 1H, *J*=10.5, 4.5 Hz, C*H*H), 3.63 (dd, 1H, *J*=10.5, 4.5 Hz, C*H*H), 0.90 (s, 9H, 3×CH_3), 0.091 (s, 3H, CH_3), 0.089 (s, 3H, CH_3). LRMS (ES+): *m/z* (%) 380 (100) [^{79}Br M+H]$^+$, 382 (93) [^{81}Br M+H]$^+$. Rf (silica, 20:80 EtOAc:hexane): 0.38.

(*S*)-2-bromo-1-(3-((*tert*-butyldimethylsilyl)oxy)-2-((4-(trifluoromethoxy)benzyl)oxy)propyl)-4-nitro-1*H*-imidazole (4) (2).

NaH (60% suspension in oil, 655 mg, 16.4 mmol) was added to a solution of alcohol **3** (4.15 g, 10.9 mmol), 4-(trifluoromethoxy)benzyl bromide (5.57 g, 21.8 mmol) and TBAI (403 mg, 1.09 mmol) in anhydrous DMF (110 ml) at -50°C. The reaction was allowed to warm to 0°C, stirred for 15 min and subsequently stirred at room temperature for a further 16 h. The reaction was then poured into a EtOAc/satd. aq. $NaHCO_3$ mixture (1:1, 400 ml), the layers separated, and the aq. extracted with additional EtOAc (3×200 ml). The combined EtOAc layers were dried over $MgSO_4$, filtered and the solvent removed under reduced pressure. The crude product was purified by silica column chromatography (120 g silica, EtOAc/Hexane 0:100 → 50:50) to give the benzyl ether **4** as a yellow oil (3.08 g, 51%). ^{1}H NMR (500 MHz, $CDCl_3$): δ 7.85 (s, 1H, ArH), 7.21-7.16 (m, 4H, 4×ArH), 4.58 (d, 1H, *J*=12.0 Hz, C*H*H), 4.41 (d, 1H, *J*=12.0 Hz, C*H*H), 4.36 (dd, 1H, *J*=14.5, 3.5 Hz, C*H*H), 4.02 (dd, 1H, *J*=14.5, 8.0 Hz, C*H*H), 3.75 (dd, 1H, *J*=10.5, 4.5 Hz, C*H*H), 3.71-3.66 (m, 1H, CH), 3.61 (dd, 1H, *J*=10.5, 6.5 Hz, C*H*H), 0.92 (s, 9H, 3×CH_3), 0.09 (s, 3H, CH_3), 0.08 (s, 3H, CH_3). ^{19}F NMR (470 MHz, $CDCl_3$): δ -57.9 (CF_3). Rf (silica, 20:80 EtOAc:hexane): 0.53.

(*S*)-3-(2-bromo-4-nitro-1*H*-imidazol-1-yl)-2-((4-(trifluoromethoxy)benzyl)oxy)propan-1-ol (5) (2).

A solution of TBAF (1 M in THF, 13.2 ml) was added to a solution of silyl ether **4** (2.44 g, 4.40 mmol) in anhydrous THF (45 ml) and stirred for 45 min at room temperature. The reaction mixture was then directly purified by silica column chromatography (120 g silica, MeOH/CH_2Cl_2 0:100 $\rightarrow$ 15:85) to give the title compound as a clear oil (1.63 g, 84%). ^{1}H NMR (500 MHz, $CDCl_3$): δ 7.86 (s, 1H, ArH), 7.24-7.81 (m, 4H, 4×ArH), 4.62 (d, 1H, *J*=12.0 Hz, C*H*H), 4.45 (d, 1H, *J*=12.0 Hz, C*H*H), 4.26 (dd, 1H, *J*=15.0, 4.5 Hz, C*H*H), 4.19 (dd, 1H, *J*=15.0, 8.0 Hz, C*H*H), 3.85 (dd, 1H, *J*=11.5, 5.0 Hz, C*H*H), 3.80-3.76 (m, 1H, CH), 3.70 (dd, 1H, *J*=11.5, 3.5 Hz, C*H*H). ^{19}F NMR (470 MHz, $CDCl_3$): δ -57.8 (CF_3). LRMS (ES+): *m/z* (%) 440 (100) [^{79}Br M+H]$^+$, 442 (93) [^{81}Br M+H]$^+$.

(*S*)-2-nitro-6-(4-(trifluoromethoxy)benzyloxy)-6,7-dihydro-5*H*-imidazo[2,1-*b*][1,3]oxazine ((*S*)-PA-824, 6).

Method 1 (Scheme S1) (3).

NaH (60% suspension in oil, 88 mg, 2.20 mmol) was added to a solution of alcohol **5** (554 mg, 1.26 mmol) in anhydrous DMF (25 ml) at 0°C and stirred for 15 min. The reaction was then allowed to warm to room temperature and stirred for a further 2 h. The reaction was cooled to -50°C prior to the addition of satd. aq. $NaHCO_3$ (15 ml) and satd. aq. NaCl (10 ml). The crude mixture was then extracted with CH_2Cl_2 (3×50 ml) followed by EtOAc (2×50 ml). The combined organic layers were subsequently dried over $MgSO_4$, filtered and the solvent removed under reduced pressure to give an orange solid. The crude product was purified by silica column chromatography (80 g silica, EtOAc/Hexane 0:100 $\rightarrow$ 100:0) to give (*S*)-PA-824 (**6**) as a pale yellow solid (251 mg, 56%).

Method 2 (Scheme S2) (4).

NaH (60% in oil, 86 mg, 3.6 mmol) was added to a solution of (*S*)-2-nitro-6,7-dihydro-5*H*-imidazo[2,1-*b*][1,3]oxazin-6-ol (**12**) (555 mg, 3 mmol), tetrabutylammonium iodide (111 mg, 0.3 mmol) and 4-(trifluoromethoxy)benzyl bromide (918 mg, 3.6 mmol) in anhydrous DMF (25 ml) at -50°C and stirred for 30 min. The reaction was subsequently allowed to warm to room temperature and stirred for a further 16 h. Workup was initiated by the dropwise addition of methanol (2 ml), followed by a solution of satd. aq. NH_4Cl (100 ml). The mixture was then extracted with CH_2Cl_2 (4×100 ml), the combined CH_2Cl_2 layers dried over $MgSO_4$, filtered and the solvent removed under reduced pressure. The crude product was purified by flash column

chromatography (60 g silica, EtOAc/Hexane 0:100 → 100:0) to give an off-white solid (789 mg, 73%).

^{1}H NMR (500 MHz, DMSO-d_6): δ 8.04 (s, 1H, ArH), 7.45-7.43 (m, 2H, AA'BB' 2×ArH), 7.36-7.33 (m, 2H, AA'BB' 2×ArH), 4.71-4.65 (m, 3H, C*H*H & CH_2), 4.47 (d, 1H, *J*=12.0 Hz, C*H*H), 4.29-4.21 (m, 3H, CH & CH_2). ^{19}F NMR (470 MHz, DMSO-d_6): δ -56.8 (CF_3). ^{13}C NMR (125 MHz, DMSO-d_6): δ 147.7 (C), 147.1 (C), 142.1 (C), 137.3 (C), 129.4 (CH), 120.9 (CH), 118.0 (CH), 68.7 (CH_2), 67.8 (CH_2), 66.6 (CH), 46.7 (CH_2) [Note, the CF_3 resonance is not visible]. LRMS (ES+): *m/z* (%) 360 (100) $[M+H]^+$, 719 (75) $[2M+H]^+$. HRMS (ES+): calcd. for $C_{14}H_{13}F_3N_3O_5$ $[M+H]^+$ 360.0802, found 360.0794 (2.10 ppm). $[\alpha]_D^{20}$ = -46.7 (*c* 1.00, MeOH), literature (1) $[\alpha]_D^{20}$ = -44.7 (*c* 1.00, MeOH). Rf (silica, 100% EtOAc): 0.33.

Synthesis of (*R*)-PA-824 (11).

(*R*)-PA-824 (**11**) was prepared in five steps from 2-bromo-4-nitro-imidazole (**1**) (Scheme S1).

(*R*)-1-(2-bromo-4-nitro-1H-imidazol-1-yl)-3-((*tert*-butyldimethylsilyl)oxy)propan-2-ol (8). (1)

Neat *tert*-butyl(dimethyl)silyl (2*R*)-2-oxiranylmethyl ether (**7**) (8.46 g, 45 mmol), was added to a solution of 2-bromo-4-nitro-imidazole (**1**) (5.76 g, 30 mmol) and K_2CO_3 (580 mg, 4.2 mmol) in anhydrous EtOH (150 ml) and stirred at 78°C for 3 h, after which the solvent was removed under reduced pressure. The resultant crude mixture was partitioned between EtOAc and satd. aq. $NaHCO_3$ (1:1, 500 ml), the layers separated and the aq. extracted with EtOAc (3×250 ml). The combined EtOAc layers were dried over $MgSO_4$, filtered and the solvent removed under reduced pressure. The crude product was purified by silica column chromatography (240 g silica, EtOAc/Hexane 10:90 → 40:60) to give the title compound as a white solid (5.66 g, 50%). ^{1}H NMR (500 MHz, $CDCl_3$): δ 7.97 (s, 1H, ArH), 4.19 (dd, 1H, *J*=13.0, 2.0 Hz, C*H*H), 4.04-3.96 (m, 2H, CH & C*H*H), 3.72 (dd, 1H, *J*=10.5, 4.5 Hz, C*H*H), 3.59 (dd, 1H, *J*=10.5, 5.0 Hz, C*H*H), 2.58 (d, 1H, *J*=5.5 Hz, OH), 0.92 (s, 9H, 3×CH_3), 0.110 (s, 3H, CH_3), 0.107 (s, 3H, CH_3). LRMS (ES+): *m/z* (%) 380 (95) [^{79}Br $M+H]^+$, 382 (100) [^{81}Br $M+H]^+$, 761 (18) [^{79}Br ^{81}Br $2M+H]^+$, 776 (16) [$^{79}Br_2$ $2M+NH_4]^+$, 778 (34) [^{79}Br ^{81}Br $2M+NH_4]^+$, 780 (21) [$^{81}Br_2$ $2M+NH_4]^+$. Rf (silica, 30:70 EtOAc:hexane): 0.47.

(*R*)-2-bromo-1-(3-((tert-butyldimethylsilyl)oxy)-2-((4-(trifluoromethoxy)benzyl)oxy)propyl)-4-nitro-1*H*-imidazole (9). (2)

NaH (60% suspension in oil, 570 mg, 14.3 mmol) was added to a solution of alcohol **8** (3.61 g, 9.5 mmol), 4-(trifluoromethoxy)benzyl bromide (4.85 g, 19 mmol) and TBAI (351 mg, 0.95 mmol) in anhydrous DMF (95 ml) at -50°C. The reaction was allowed to warm to 0°C, stirred for 15 min and subsequently stirred at room temperature for a further 16 h. The reaction was then poured into a EtOAc/satd. aq. $NaHCO_3$ mixture (1:1, 500 ml), the layers separated, and the aq. extracted with additional EtOAc (3×250 ml). The combined EtOAc layers were dried over $MgSO_4$, filtered and the solvent removed under reduced pressure. The crude product was purified by silica column chromatography (120 g silica, EtOAc/Hexane 10:90 → 50:50) to give the benzyl ether **9** as a yellow oil (2.71 g, 52%). ^{1}H NMR (500 MHz, $CDCl_3$): δ 7.84 (s, 1H, ArH), 7.20-7.18 (m, 2H, AA'BB', 2×ArH), 7.15-7.12 (m, 2H, AA'BB', 2×ArH), 4.58 (d, 1H, *J*=12.0 Hz, C*H*H), 4.40 (d, 1H, *J*=12.0 Hz, C*H*H), 4.30 (dd, 1H, *J*=14.5, 3.0 Hz, C*H*H), 4.03 (dd, 1H, *J*=14.5, 8.0 Hz, C*H*H), 3.74 (dd, 1H, *J*=10.5, 4.0 Hz, C*H*H), 3.71-3.66 (m, 1H, CH), 3.61 (dd, 1H, *J*=10.5, 6.5 Hz, C*H*H), 0.90 (s, 9H, 3×CH_3), 0.07 (s, 3H, CH_3), 0.06 (s, 3H, CH_3). ^{19}F NMR (470 MHz, $CDCl_3$): δ -57.9 (CF_3). ^{13}C NMR (125 MHz, $CDCl_3$): δ 149.0 (C), 147.1 (C), 135.8 (C), 129.3 (CH), 123.0 (CH), 121.1 (CH), 120.5 (C), 120.4 (q, *J*=256 Hz, CF_3), 77.5 (CH), 71.6 (CH_2), 61.6 (CH_2), 50.2 (CH_2), 25.8 (CH_3), 18.2 (C), -5.5 (CH_3). LRMS (ES+): *m/z* (%) 554 (100) [^{79}Br M+H]$^+$, 556 (93) [^{81}Br M+H]$^+$. Rf (silica, 20:80 EtOAc:hexane): 0.39.

(*R*)-3-(2-bromo-4-nitro-1*H*-imidazol-1-yl)-2-((4-(trifluoromethoxy)benzyl)oxy)propan-1-ol (10). (2)

A solution of TBAF (1 M in THF, 14.6 ml) was added to a solution of silyl ether **9** (2.69 g, 4.85 mmol) in anhydrous THF (50 ml) and stirred for 45 min at room temperature. The reaction mixture was then directly purified by silica column chromatography (120 g silica, MeOH/CH_2Cl_2 0:100 → 15:85) to give the title compound as a pale yellow oil (1.95 g, 91%). ^{1}H NMR (500 MHz, $CDCl_3$): δ 7.87 (s, 1H, ArH), 7.22-7.20 (m, 2H, AA'BB', 2×ArH), 7.17-7.14 (m, 2H, AA'BB', 2×ArH), 4.61 (d, 1H, *J*=12.0 Hz, C*H*H), 4.43 (d, 1H, *J*=12.0 Hz, C*H*H), 4.26 (dd, 1H, *J*=14.5, 4.0 Hz, C*H*H), 4.18 (dd, 1H, *J*=14.5, 8.0 Hz, C*H*H), 3.83 (dd, 1H, *J*=11.5, 5.0 Hz, C*H*H), 3.79-3.76 (m, 1H, CH), 3.71 (dd, 1H, *J*=11.5, 3.5 Hz, C*H*H). ^{19}F NMR (282 MHz, $CDCl_3$): δ -57.8 (CF_3). LRMS (ES+): *m/z* (%) 440 (100) [^{79}Br M+H]$^+$, 442 (95) [^{81}Br M+H]$^+$. Rf (silica, 50:50 EtOAc:hexane): 0.11.

(*R*)-2-nitro-6-(4-(trifluoromethoxy)benzyloxy)-6,7-dihydro-5*H*-imidazo[2,1-*b*][1,3]oxazine ((*R*)-PA-824, 11). (3)

NaH (60% suspension in oil, 302 mg, 7.55 mmol) was added to a solution of alcohol **10** (1.90 g, 4.32 mmol) in anhydrous DMF (85 ml) at 0°C and stirred for 15 min. The reaction was then allowed to warm to room temperature and stirred for a further 2 h. The reaction was cooled to -50°C prior to the addition of satd. aq. $NaHCO_3$ (15 ml) and satd. aq. NaCl (10 ml). The crude mixture was then extracted with CH_2Cl_2 (3×50 ml) followed by EtOAc (2×50 ml). The combined organic layers were subsequently dried over $MgSO_4$, filtered and the solvent removed under reduced pressure to give an orange solid. The crude product was purified by silica column chromatography (120 g silica, EtOAc/Hexane 0:100 → 100:0) to give (*R*)-PA-824 (**11**) as an off-white solid (464 mg, 30%). ^1H NMR (500 MHz, DMSO-d_6): δ 8.02 (s, 1H, ArH), 7.45-7.43 (m, 2H, AA'BB' 2×ArH), 7.35-7.33 (m, 2H, AA'BB' 2×ArH), 4.71-4.65 (m, 3H, C*H*H & CH_2), 4.47 (d, 1H, *J*=12.0 Hz, C*H*H), 4.29-4.22 (m, 3H, CH & CH_2). ^{19}F NMR (470 MHz, DMSO-d_6): δ -56.8 (CF_3). LRMS (ES+): *m/z* (%) 360 (100) $[M+H]^+$. HRMS (ES+): calcd. for $C_{14}H_{13}F_3N_3O_5$ $[M+H]^+$ 360.0802, found 360.0812 (-2.80 ppm). $[\alpha]_D^{20}$ = +46.1 (*c* 1.00, MeOH). Rf (silica, 100% EtOAc): 0.49.

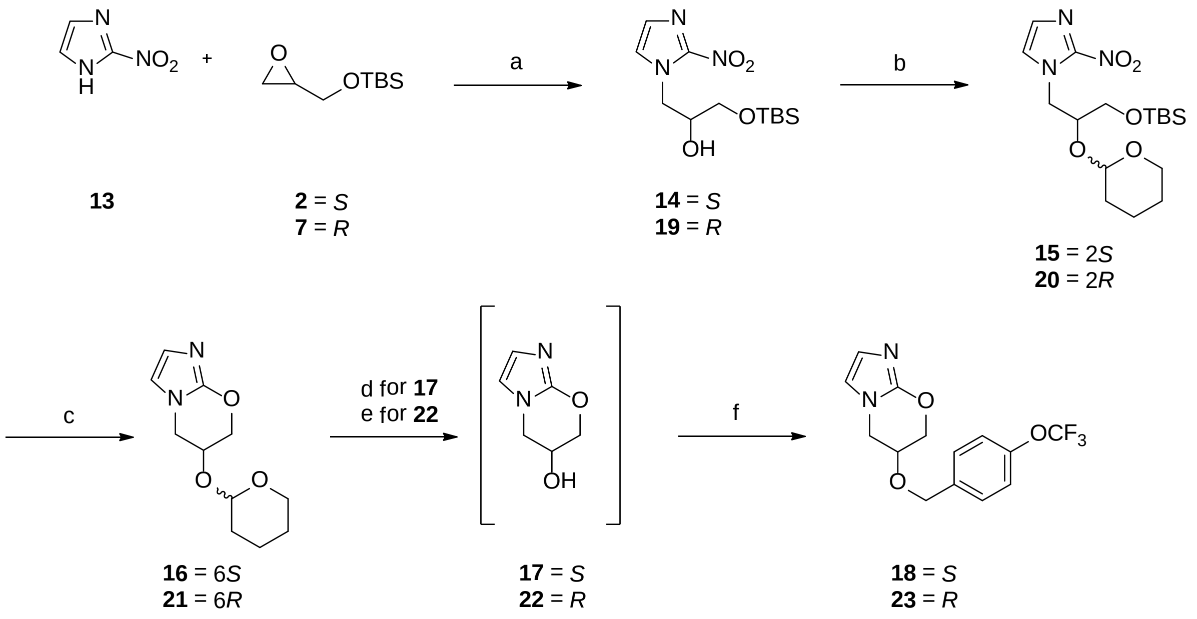

Scheme S3: Synthesis of (*S*)- (18) and (*R*)-*des*-nitro-PA-824 (**23**). Reagents and conditions: (a) K_2CO_3, EtOH, 78°C, 2-3 h, 32-49%; (b) 3,4-dihydropyran, PPTS, CH_2Cl_2, RT, 16 h, 71-90%; (c) TBAF, THF, 125°C, 15 h, 73-84%; (d) AcOH, H_2O, THF, 60°C, 16 h; (e) H_3CSO_3H, MeOH, RT, 24 h; (f) NaH, TBAI, *p*-OCF_3-BnBr, DMF, -42°C →RT, 16 h, 24-29%.

Synthesis of (*S*)-des-nitro-PA-824 (18).

(*S*)-des-nitro-PA-824 (**18**) was prepared in 5 steps according to the method of Singh *et al* (4) (Scheme S3).

(*S*)-1-((*tert*-butyldimethylsilyl)oxy)-3-(2-nitro-1*H*-imidazol-1-yl)propan-2-ol (14). Neat *tert*-butyldimethylsilyl (*S*)-glycidyl ether (1.41 g, 7.5 mmol) was added to a solution of 2-nitroimidazole (565 mg, 5 mmol) and K_2CO_3 (97 mg, 0.7 mmol) in anhydrous EtOH (6 ml) and stirred for 3 h at 78°C, after which the solvent was removed under reduced pressure. CH_2Cl_2 (20 ml) was then added to the residue, the resultant suspension was filtered, dried over $MgSO_4$, re-filtered and evaporated under reduced pressure. The crude product was purified by silica column chromatography (80 g silica, EtOAc/hexane 0:100 → 75:25) to give the title compound as an off-white solid (745 mg, 49%). ^{1}H NMR (500 MHz, $CDCl_3$): δ 7.19 (d, 1H, *J*=1.0 Hz, ArH), 7.06 (d, 1H, *J*=1.0 Hz, ArH), 4.68 (dd, 1H, *J*=14.0, 3.5 Hz, C*H*H), 4.32 (dd, 1H, *J*=14.0, 8.5 Hz, C*H*H), 4.06-4.01 (m, 1H, CH), 3.70 (dd, 1H, *J*=10.5, 5.0 Hz, C*H*H), 3.62 (dd, 1H, *J*=10.5, 5.0 Hz, C*H*H), 3.03 (d, 1H, *J*=6.0 Hz, OH), 0.90 (s, 9H, 3×CH_3), 0.08 (s, 6H, 2×CH_3). ^{13}C NMR (125 MHz, $CDCl_3$): δ 127.9 (CH), 127.3 (CH), 70.4 (CH), 64.2 (CH_2), 52.5 (CH_2), 25.8 (CH_3), 18.2 (C). [Note, one quaternary carbon resonance is not visible]. LRMS (ES+): *m/z* (%) 302 (71) $[M+H]^+$, 603 (100) $[2M+H]^+$. Rf (silica, 50:50 EtOAc:hexane): 0.39.

1-((2*S*)-3-((tert-butyldimethylsilyl)oxy)-2-((tetrahydro-2*H*-pyran-2-yl)oxy)propyl)-2-nitro-1*H*-imidazole (15). 3,4-Dihydro-2*H*-pyran was added to a solution of alcohol **14** (754 mg, 2.5 mmol) and PPTS (924 mg, 3.75 mmol) in anhydrous CH_2Cl_2 (10 ml) and stirred at room temperature for 16 h. The workup was initiated by the addition of satd. aq. $NaHCO_3$ (20 ml), after which the layers were separated and the aq. layer further extracted with CH_2Cl_2 (3×20 ml). The combined CH_2Cl_2 layers were washed with satd. aq. NaCl (50 ml), dried over $MgSO_4$, filtered and the solvent removed under reduced pressure. The crude product was purified by silica column chromatography (40 g silica, EtOAc/hexane 0:100 → 100:00) to give the THP protected alcohol **15** as a clear oil (684 mg, 71%). Note, the product is prepared as a 1:1 mixture of diasteroisomers. ^{1}H NMR (500 MHz, $CDCl_3$): δ 7.18 (d, 1H, *J*=1.0 Hz, ArH), 7.12 (d, 1H, *J*=1.0 Hz, ArH), 7.10 (d, 1H, *J*=1.0 Hz, ArH), 7.09 (d, 1H, *J*=1.0 Hz, ArH), 4.94 (dd, 1H, *J*=14.0, 3.5 Hz, C*H*H), 4.76 (dd, 1H, *J*=14.0, 3.5 Hz, C*H*H), 4.65-4.63 (m, 1H, CH), 4.44 (dd, 1H, *J*=14.0, 8.0 Hz, C*H*H), 4.32 (dd, 1H, *J*=14.0, 7.0 Hz, C*H*H), 4.28-4.26 (m, 1H, CH), 4.08-4.05 (m, 1H, CH), 3.93-3.89 (m, 1H, CH), 3.83-3.78 (m, 2H, 2×C*H*H), 3.65 (dd, 1H, *J*=11.0, 4.5 Hz, C*H*H), 3.60 (dd, 1H, *J*=11.0, 5.5 Hz, C*H*H), 3.47 (dd, 1H, *J*=10.5, 7.5 Hz, C*H*H), 3.30-3.26 (m, 1H, C*H*H), 3.22-3.18 (m, 1H, C*H*H), 1.72-1.34 (m, 12H, 6×CH_2), 0.91 (s, 9H, 3×CH_3), 0.89

(s, 9H, 3×CH_3), 0.08 (s, 6H, 2×CH_3), 0.06 (s, 6H, 2×CH_3). LRMS (ES+): *m/z* (%) 302 (23) $[M\text{-}THP+H]^+$, 386 (100) $[M+H]^+$. Rf (silica, 50:50 EtOAc:hexane): 0.79.

(6*S*)-6-((tetrahydro-2*H*-pyran-2-yl)oxy)-6,7-dihydro-5H-imidazo[2,1-*b*][1,3]oxazine (16). A solution of TBAF (1 M in THF, 2.25 ml) was added to a solution of silyl ether **15** (289 mg, 0.75 mmol) in anhydrous THF (7.75 ml) and heated to 125°C under microwave irradiation for 15 h. Subsequently, the reaction solvent was removed under reduced pressure and the resultant mixture partitioned between satd. aq. $NaHCO_3/CH_2Cl_2$ (1:1, 20 ml). The biphasic mixture was then separated and the aq. layer further extracted with CH_2Cl_2 (3×10 ml). The combined CH_2Cl_2 layers were then dried over $MgSO_4$, filtered and the solvent removed under reduced pressure. The crude product was purified using silica column chromatography (40 g silica, MeOH/EtOAc 0:100 $\rightarrow$ 20:80) to give the title compound as a brown oil (141 mg, 84%). Note, the product is prepared as a 1:1 mixture of diasteroisomers. ^1H NMR (500 MHz, $CDCl_3$): δ 6.63-6.62 (m, 2H, 2×ArH), 6.46 (d, 1H, *J*=1.5 Hz, ArH), 6.45 (d, 1H, *J*=1.5 Hz, ArH), 4.81-4.77 (m, 2H, 2×CH), 4.41-4.32 (m, 3H, C*H*H & CH_2), 4.26-4.19 (m, 3H, C*H*H & CH_2), 4.13 (dd, 1H, *J*=12.5, 4.5 Hz, C*H*H), 4.08-4.02 (m, 2H, 2×C*H*H), 3.93-3.83 (m, 2H, 2×C*H*H), 3.77-3.72 (m, 1H, C*H*H), 3.54-3.47 (m, 2H, CH_2), 1.80-1.46 (m, 12H, 6×CH_2). ^{13}C NMR (125 MHz, $CDCl_3$): δ 149.3 (C), 149.1 (C), 124.43 (CH), 124.35 (CH), 114.1 (CH), 114.0 (CH), 98.2 (CH), 97.9 (CH), 69.4 (CH_2), 67.1 (CH_2), 65.7 (CH), 65.2 (CH), 62.8 (CH_2), 62.5 (CH_2), 47.7 (CH_2), 46.2 (CH_2), 30.5 (CH_2), 30.4 (CH_2), 25.2 (CH_2), 19.2 (CH_2), 19.0 (CH_2). [Note, one CH_2 carbon resonance is not visible, ^1H-^{13}C HSQC shows that two CH_2 resonances are coincident at 25.2 ppm]. LRMS (ES+): *m/z* (%) 225 (100) $[M+H]^+$, 449 (15) $[2M+H]^+$. Rf (silica, $KMnO_4$ stain, 10:90 EtOH:EtOAc): 0.23.

(*S*)-6,7-dihydro-5*H*-imidazo[2,1-*b*][1,3]oxazin-6-ol (17). AcOH (4 ml) was added to a solution of THP ether **16** (112 mg, 0.5 mmol) in THF/water (2:1, 3 ml) and stirred at 60°C for 16 h after which the solvent was removed under reduced pressure. The crude reaction mixture was then re-dissolved in EtOH/toluene (1:1 20 ml) and again evaporated *in vacuo* to give the crude product (60 mg, 86%) which was subsequently used without further purification.

(*S*)-6-((4-(trifluoromethoxy)benzyl)oxy)-6,7-dihydro-5*H*-imidazo[2,1-*b*][1,3]oxazine ((*S*)-*des*-nitro-PA-824, 18). NaH (60% in oil, 10 mg, 0.24 mmol) was added to a solution of TBAI (7.4 mg, 0.02 mmol), alcohol **17** (28 mg, 0.2 mmol) and 4-(trifluoromethoxy)benzyl bromide (61 mg, 0.24 mmol) in anhydrous DMF (4 ml) at -50°C. The reaction was allowed to warm to room temperature and then stirred for a further 16 h. The reaction was worked up by the addition of water (0.5 ml) and MeCN (0.5 ml). The crude reaction mixture was then filtered and purified directly by reverse phase HPLC to give (*S*)-*des*-nitro-PA-824 (**18**) as an off-white solid (18 mg,

29%). ^{1}H NMR (500 MHz, DMSO-d_6): δ 7.46-7.43 (m, 2H, AA'BB' 2×ArH), 7.35-7.33 (m, 2H, AA'BB' 2×ArH), 6.69 (s, 1H, *J*=1.5 Hz, ArH), 6.47 (s, 1H, *J*=1.5 Hz, ArH), 4.68 (d, 1H, *J*=12.5 Hz, C*H*H), 4.64 (d, 1H, *J*=12.5 Hz, C*H*H), 4.53-4.50 (m, 1H, C*H*H), 4.31-4.29 (m, 1H, C*H*H), 4.14-4.07 (m, 3H, CH & CH_2). ^{19}F NMR (470 MHz, DMSO-d_6): δ -56.8 (CF_3). ^{13}C NMR (125 MHz, DMSO-d_6): δ 148.5 (C), 147.7 (C), 137.6 (C), 129.4 (CH), 123.0 (CH), 121.0 (CH), 114.6 (CH), 68.7 (CH_2), 67.4 (CH), 66.9 (CH_2), 45.8 (CH_2). [Note, the CF_3 resonance is not visible]. LRMS (ES+): *m/z* (%) 315 (100) $[M+H]^+$.

Synthesis of (*R*)-des-nitro-PA-824 (23).

(*R*)-des-nitro-PA-824 (**23**) was prepared in 5 steps using the methodology described in Singh *et al* (3) (Scheme S3).

(*R*)-1-((*tert*-butyldimethylsilyl)oxy)-3-(2-nitro-1*H*-imidazol-1-yl)propan-2-ol (19). Neat *tert*-butyldimethylsilyl (*R*)-glycidyl ether (**7**) (2.83 g, 15 mmol) was added to a solution of 2-nitroimidazole (**13**) (1.13 g, 10 mmol) and K_2CO_3 (193 mg, 1.4 mmol) in anhydrous EtOH (6 ml) and stirred for 2 h at 78°C, after which the solvent was removed under reduced pressure. CH_2Cl_2 (50 ml) was then added to the residue, the resultant suspension was filtered and the solid washed with CH_2Cl_2 (3 × 50 ml). The combined filtrates were dried over $MgSO_4$, re-filtered and evaporated under reduced pressure. The crude product was purified by silica column chromatography (80 g silica, EtOAc/hexane 10:100 → 100:00) to give the title compound as an off-white solid (973 mg, 32%). ^{1}H NMR (500 MHz, $CDCl_3$): δ 7.20 (d, 1H, *J*=1.0 Hz, ArH), 7.15 (d, 1H, *J*=1.0 Hz, ArH), 4.66 (dd, 1H, *J*=14.0, 3.0 Hz, C*H*H), 4.37 (dd, 1H, *J*=14.0, 8.0 Hz, C*H*H), 4.06-4.01 (m, 1H, CH), 3.76 (dd, 1H, *J*=10.5, 4.0 Hz, C*H*H), 3.59 (dd, 1H, *J*=10.5, 5.0 Hz, C*H*H), 2.50 (d, 1H, *J*=6.0 Hz, OH), 0.92 (s, 9H, 3×CH_3), 0.10 (s, 6H, 2×CH_3). LRMS (ES+): *m/z* (%) 302 (100) $[M+H]^+$. Rf (silica, 50:50 EtOAc:hexane): 0.48.

1-((2*R*)-3-((tert-butyldimethylsilyl)oxy)-2-((tetrahydro-2*H*-pyran-2-yl)oxy)propyl)-2-nitro-1*H*-imidazole (20). 3,4-Dihydro-2*H*-pyran (2.72 g, 32.3 mmol) was added to a solution of alcohol **19** (973 mg, 3.23 mmol) and PPTS (1.22 g, 4.84 mmol) in anhydrous CH_2Cl_2 (30 ml) and stirred at room temperature for 16 h. The workup was initiated by the addition of satd. aq. $NaHCO_3$ (30 ml), after which the layers were separated and the aq. layer further extracted with CH_2Cl_2 (3×30 ml). The combined CH_2Cl_2 layers were dried over $MgSO_4$, filtered and the solvent removed under reduced pressure. The crude product was purified by silica column chromatography (40 g silica, EtOAc/hexane 10:90 → 60:40) to give the THP protected alcohol **20** as a clear oil (1.12 g, 90%). Note, the product is prepared as a 1:1 mixture of diastereoisomers. ^{1}H NMR (500 MHz, $CDCl_3$): δ 7.19 (d, 1H, *J*=1.0 Hz, ArH), 7.13 (d, 1H,

J=1.0 Hz, ArH), 7.12 (d, 1H, *J*=1.0 Hz, ArH), 7.11 (d, 1H, *J*=1.0 Hz, ArH), 4.96 (dd, 1H, *J*=14.0, 3.5 Hz, C*H*H), 4.78 (dd, 1H, *J*=14.0, 3.5 Hz, C*H*H), 4.68-4.64 (m, 1H, CH), 4.45 (dd, 1H, *J*=14.0, 8.0 Hz, C*H*H), 4.33 (dd, 1H, *J*=14.0, 7.0 Hz, C*H*H), 4.29-4.27 (m, 1H, CH), 4.10-4.06 (m, 1H, CH), 3.95-3.90 (m, 1H, CH), 3.85-3.79 (m, 2H, 2×C*H*H), 3.65 (dd, 1H, *J*=11.0, 4.5 Hz, C*H*H), 3.61 (dd, 1H, *J*=11.0, 5.5 Hz, C*H*H), 3.48 (dd, 1H, *J*=10.5, 8.0 Hz, C*H*H), 3.46-3.42 (m, 1H, C*H*H), 3.31-3.27 (m, 1H, C*H*H), 3.23-3.18 (m, 1H, C*H*H), 1.74-1.36 (m, 12H, 6×CH_2), 0.93 (s, 9H, 3×CH_3), 0.91 (s, 9H, 3×CH_3), 0.09 (s, 6H, 2×CH_3), 0.07 (s, 6H, 2×CH_3). ^{13}C NMR (125 MHz, $CDCl_3$): δ 145.4 (C), 127.7 (CH), 127.5 (CH), 127.4 (CH), 127.0 (CH), 100.2 (CH), 96.9 (CH), 76.9 (CH), 73.6. (CH), 63.1 (CH_2), 63.0 (CH_2), 62.5 (CH_2), 61.8 (CH_2), 51.2 (CH_2), 50.5 (CH_2), 30.6 (CH_2), 30.4 (CH_2), 25.84 (CH_3), 25.80 (CH_3), 25.2 (CH_2), 25.0 (CH_2), 19.7 (CH_2), 19.5 (CH_2), 18.23 (C), 18.22 (C), -5.45 (CH_3), -5.48 (CH_3) [Note, one quaternary carbon resonance is not visible, ^{1}H-^{13}C HMBC shows that two C resonances are coincident at 145.4 ppm]. LRMS (ES+): *m/z* (%) 302 (15) $[M-THP+H]^+$, 372 (11) $[M-CH_3+H]^+$, 386 (100) $[M+H]^+$, 408 (30) $[M+Na]^+$. Rf (silica, 25:75 EtOAc:hexane): 0.47.

(6*R*)-6-((tetrahydro-2*H*-pyran-2-yl)oxy)-6,7-dihydro-5H-imidazo[2,1-*b*][1,3]oxazine (21). A solution of TBAF (1 M in THF, 2.25 ml) was added to a solution of silyl ether **20** (289 mg, 0.75 mmol) in anhydrous THF (7.75 ml) and heated to 125°C under microwave irradiation for 15 h. Subsequently, the reaction solvent was removed under reduced pressure and the resultant mixture partitioned between satd. aq. $NaHCO_3/CH_2Cl_2$ (1:1, 50 ml). The biphasic mixture was then separated and the aq. layer further extracted with CH_2Cl_2 (3×25 ml). The combined CH_2Cl_2 layers were then dried over $MgSO_4$, filtered and the solvent removed under reduced pressure. The crude product was purified using silica column chromatography (40 g silica, MeOH/EtOAc 0:100 → 20:80) to give the title compound as a pale brown oil (122 mg, 73%). Note, the product is prepared as a 1:1 mixture of diastereoisomers. ^{1}H NMR (500 MHz, $CDCl_3$): δ 6.65-6.63 (m, 2H, 2×ArH), 6.47 (d, 1H, *J*=2.0 Hz, ArH), 6.46 (d, 1H, *J*=1.5 Hz, ArH), 4.82-4.81 (m, 1H, CH), 4.79-4.78 (m, 1H, CH), 4.42-4.33 (m, 3H, C*H*H & CH_2), 4.27-4.20 (m, 3H, C*H*H & 2×CH), 4.14 (ddd, 1H, *J*=12.5, 4.5, 0.5 Hz, C*H*H), 4.08-4.01 (m, 2H, 2×C*H*H), 3.92 (ddd, 1H, *J*=12.5, 4.0, 1.5 Hz, C*H*H), 3.89-3.84 (m, 1H, C*H*H), 3.77-3.73 (m, 1H, C*H*H), 3.55-3.48 (m, 2H, CH_2), 1.80-1.44 (m, 12H, 6×CH_2). ^{13}C NMR (125 MHz, $CDCl_3$): δ 149.3 (C), 149.1 (C), 124.34 (CH), 124.28 (CH), 114.1 (CH), 114.0 (CH), 98.1 (CH), 97.8 (CH), 69.4 (CH_2), 67.0 (CH_2), 65.6 (CH), 65.2 (CH), 62.8 (CH_2), 62.5 (CH_2), 47.7 (CH_2), 46.1 (CH_2), 30.5 (CH_2), 30.4 (CH_2), 25.2 (CH_2), 19.2 (CH_2), 19.0 (CH_2). [Note, one CH_2 carbon resonance is not visible, ^{1}H-^{13}C HSQC shows that two CH_2 resonances are coincident at 25.2 ppm]. LRMS (ES+): *m/z* (%) 225 (100) $[M+H]^+$, 471 (11) $[2M+Na]^+$. Rf (silica, $KMnO_4$ stain, 10:90 EtOH:EtOAc): 0.24.

(*R*)-6,7-dihydro-5*H*-imidazo[2,1-*b*][1,3]oxazin-6-ol (22). Methanesulfonic acid (0.5 ml) was added to a solution of THP ether **21** (123 mg, 0.55 mmol) in MeOH (5 ml) and stirred at room

temperature for 24 h after which the solvent was removed under reduced pressure. The crude reaction mixture was then partitioned between satd. aq. $NaHCO_3$ and EtOAc (1:1 50 ml), the layers separated and the aq. layer subsequently extracted with EtOAc (5×25 ml). The combined EtOAc layers were dried over $MgSO_4$, filtered and evaporated under reduced pressure to give the crude product which was subsequently used without further purification.

(*R*)-6-((4-(trifluoromethoxy)benzyl)oxy)-6,7-dihydro-5*H*-imidazo[2,1-*b*][1,3]oxazine ((*R*)-des-nitro-PA-824, 18). NaH (60% in oil, 10 mg, 0.26 mmol) was added to a solution of crude alcohol **22** (30 mg, approx. 0.21 mmol) and TBAI (8 mg, 0.02 mmol), in anhydrous DMF (2 ml) at -42°C. The reaction was stirred for 30 min before the addition of 4-(trifluoromethoxy)benzyl bromide (66 mg, 0.26 mmol) after which the reaction was allowed to warm to room temperature and then stirred for a further 16 h. The reaction was worked up by the slow addition of satd. aq. NH_4Cl (2.5 ml) followed by extraction with CH_2Cl_2 (4×10 ml). The combined CH_2Cl_2 layers were then dried over $MgSO_4$, filtered and the solvent removed under reduced pressure. The crude product was then re-dissolved in DMF (0.8 ml), filtered and purified by reverse phase HPLC to give (*R*)-*des*-nitro-PA-824 (**23**) as a white solid (16 mg, 24% over two steps). ^{1}H NMR (500 MHz, DMSO-d_6): δ 7.46-7.43 (m, 2H, AA'BB' 2×ArH), 7.35-7.33 (m, 2H, AA'BB' 2×ArH), 6.69 (s, 1H, *J*=1.5 Hz, ArH), 6.47 (s, 1H, *J*=1.5 Hz, ArH), 4.68 (d, 1H, *J*=12.5 Hz, C*H*H), 4.65 (d, 1H, *J*=12.5 Hz, C*H*H), 4.53-4.50 (m, 1H, C*H*H), 4.31-4.29 (m, 1H, C*H*H), 4.14-4.07 (m, 3H, CH & CH_2). ^{19}F NMR (470 MHz, DMSO-d_6): δ -56.8 (CF_3). ^{13}C NMR (125 MHz, DMSO-d_6): δ 148.5 (C), 147.7 (C), 137.5 (C), 129.3 (CH), 122.9 (CH), 120.9 (CH), 114.5 (CH), 68.7 (CH_2), 67.4 (CH), 66.9 (CH_2), 45.7 (CH_2). [Note, the CF_3 resonance is not visible]. LRMS (ES+): *m/z* (%) 315 (100) $[M+H]^+$.

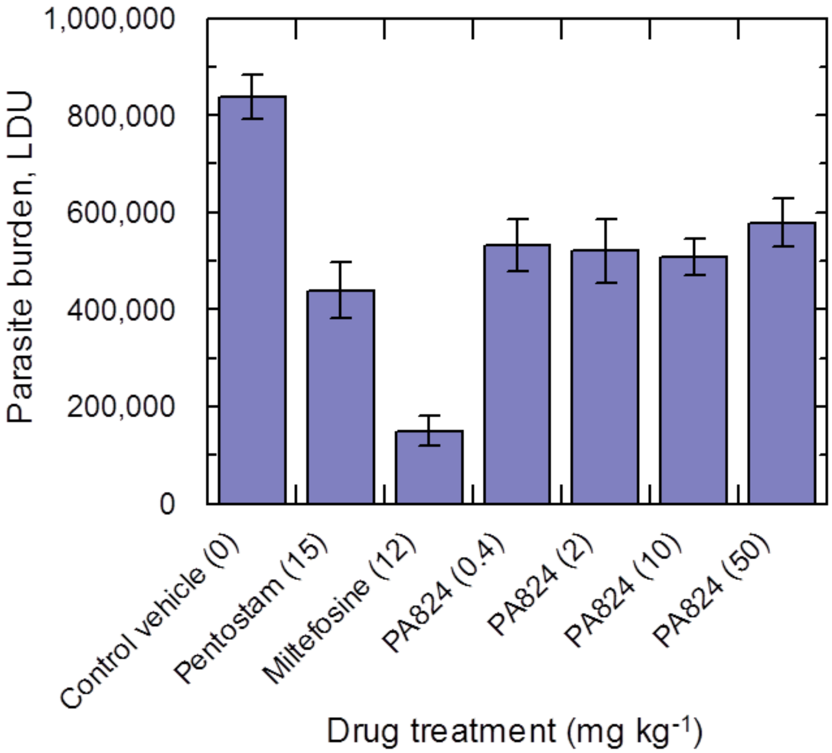

Figure S1 Effects of drug treatment on the parasite burden of mice infected with *L. donovani.* Groups of mice (five per group) infected with *L. donovani* (strain LV9) were dosed with drug vehicle (orally), Pentostam (subcutaneously), miltefosine (orally) and (*S*)-PA-824 (orally) on day 7 after infection and for the following 4 days. Miltefosine and Pentostam were dosed once daily with (*S*)-PA-824 administered twice daily. On day 14 after infection, all animals were humanely euthanized and parasite burdens were determined microscopically by examining Giemsa-stained liver smears. Parasite load is expressed in Leishman-Donovan units (LDU): mean number of amastigotes per liver cell × milligram of liver (6).

REFERENCES

1. **Orita A, Miwa K, Uehara G, and Otera J.** 2007. Integration of solventless reaction in a multi-step process: Application to an efficient synthesis of PA-824. Adv. Synth. Catal. **349**:2136-2144.

2. **Kim P, Zhang L, Manjunatha UH, Singh R, Patel S, Jiricek J, Keller TH, Boshoff HI, Barry CE, and Dowd CS.** 2009. Structure-activity relationships of antitubercular nitroimidazoles. 1. Structural features associated with aerobic and anaerobic activities of 4-and 5-nitroimidazoles. J. Med. Chem. **52**:1317-1328.

3. **Thompson AM, Sutherland HS, Palmer BD, Kmentova I, Blaser A, Franzblau SG, Wan BJ, Wang YH, Ma ZK, and Denny WA.** 2011. Synthesis and structure-activity relationships of varied ether linker analogues of the antitubercular drug (6S)-2-nitro-6-{[4-(trifluoromethoxy)benzyl]oxy}-6,7-dihydro-5H-imidazo[2,1-b][1,3]oxazine (PA-824). J. Med. Chem. **54**:6563-6585.

4. **Singh R, Manjunatha U, Boshoff HIM, Ha YH, Niyomrattanakit P, Ledwidge R, Dowd CS, Lee IY, Kim P, Zhang L, Kang SH, Keller TH, Jiricek J, and Barry CEI.** 2008. PA-824 kills nonreplicating *Mycobacterium tuberculosis* by intracellular NO release. Science **322**:1392-1395.
